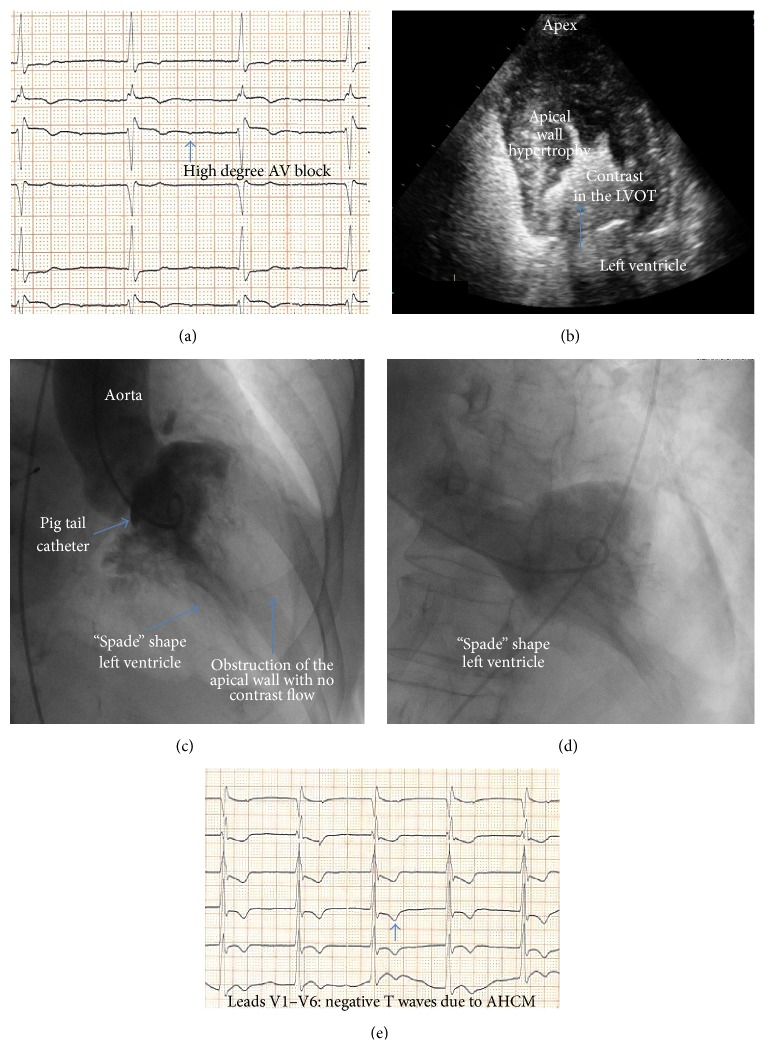# An Atypical Case of Apical Hypertrophic Cardiomyopathy: Absence of Giant T Waves in spite of Extreme Apical Wall Hypertrophy

**DOI:** 10.1155/2015/980971

**Published:** 2015-12-08

**Authors:** Elias Sanidas, Maria Bonou, Georgios Anastasiadis, Georgios Tzanis, John Barbetseas

**Affiliations:** Department of Cardiology, “Laiko” General Hospital, 17 Agiou Thoma Street, 15117 Athens, Greece

## Abstract

Apical hypertrophic cardiomyopathy is an uncommon variant of hypertrophic cardiomyopathy, with hypertrophy mainly affecting the apex of the left ventricle. We hereby describe a case of an octogenarian female patient who was randomly diagnosed with AHCM due to other comorbidities.

## 1. Introduction

Apical hypertrophic cardiomyopathy (AHCM) is a relatively rare morphologic form of hypertrophic cardiomyopathy (HCM), in which the hypertrophy of the myocardium predominantly implicates the apex of the left ventricle (LV), involving mostly Asian population [1–3]. We considered presenting this case as it underlines the unusual features of AHCM in the elderly.

## 2. Case Presentation

An 85-year-old Caucasian, smoker, and hypertensive female under chronic medical therapy with digitalis due to known permanent atrial fibrillation (AF) presented complaining of dizziness, nausea, and angina-like chest discomfort. The patient also reported episodes of syncope during the past but without any further investigation. Her electrocardiogram (ECG) showed bradyarrhythmia with a high degree atrioventricular block, due to digitalis toxicity (Figure 1(a)).

Transthoracic echocardiography indicated left atrium enlargement, a small LV with hyperdynamic contractility, and excessive hypertrophy of the apical wall of 33 mm, while contrast injection was elusive for apical AHCM (Figure 1(b)).

Coronary arteriography showed no significant stenosis. The left ventriculography revealed a typical “spade” shape ventricle (or alternatively the “ace of spade” sign) with obstruction during systole confirming the diagnosis of AHCM (Figures 1(c) and 1(d)).

Seven days after digitalis withdrawal, the ECG findings were AF with normal axis deviation and T wave inversion most prominent in the midprecordial leads (Figure 1(e)). A 24-hour Holter monitor recorded pauses of 3.5 sec with no evidence of ventricular arrhythmia and a single lead pacemaker was implanted. The octogenarian woman almost one year after her discharge remains asymptomatic.

## 3. Discussion

Our case underlines the unusual features of AHCM. This variant of nonobstructive HCM represents up to 25% of the entire HCM population in Japan and 1% to 10% in non-Asian population, while its prevalence in the elderly people remains uncertain [1, 2, 4].

Compared to all other forms of HCM, patients with AHCM tend to be much older, less symptomatic, and usually characterized by less hypertrophy. The typical features of AHCM consist of giant negative T waves on ECG and a “spade-like” configuration of the LV cavity at end-diastole on left ventriculography. Data from ECG-CMRI (cardiac magnetic resonance tomography) studies demonstrate a good correlation between negative T waves amplitude and the ratio between apical and basal septal wall thicknesses (craniocaudal asymmetry) [5–7]. However, the interesting part of this case is the absence of giant T waves. It seems that giant T waves diminish considerably with age and this is consistent with our ECG findings.

In this context, a weak correlation between the degree of apical thickness and the maximal T wave negativity has also been reported. Of note, Caucasian patients tend to have less localized involvement of the distal apex resulting in a lower frequency of the pathognomonic sign of “ace of spade” on the left ventriculography [8, 9]. Conversely, in our case there was extreme apical wall thickening of 33 mm which is found only in 1% of the patients with AHCM, predisposing for the typical appearance of giant T wave inversion on ECG. Nevertheless, severe hypertrophy >30 mm in HCM patients without other risk factors carries a low risk of sudden cardiac death. Diastolic dysfunction, left atrial enlargement with subsequent AF and progression into apical aneurysm or midventricular obstruction, has also been described as unfavorable features of the disease [10, 11].

The apical variant has a more benign prognosis than other forms of HCM as the patient in our case, but it is still associated with adverse outcome mainly in the elderly in the presence of AF. Adverse events include thromboembolic stroke, progressive heart failure myocardial infarction, and arrhythmias. AHMC in Western population patients is not frequently associated with sudden cardiac death in terms of cardiovascular mortality ranging around 0.1% [10, 12].

Small number of cases and studies documented the delayed onset of the AHCM phenotype at any age in life, resulting in late-appearing apical LV hypertrophy. Hypertension, whose prevalence is higher in the elderly cohort than the general population, has been reported that could play an added role as a trigger of AHCM. Meanwhile, this clinical entity often remains underappreciated and misdiagnosed due to lack of awareness of the condition, especially in this cohort of patients [9, 12].

## 4. Conclusions

AHCM is an uncommon clinical type of HCM. Recent data suggest that it is less benign than previously suspected, especially in Caucasian elderly women that might also present without the giant T waves on ECG. Careful care along with serious consideration of all related comorbidities and medications is needed.

## Figures and Tables

**Figure 1:**